# Cellulolytic potential of bacteria isolated from Australian marsupial herbivores

**DOI:** 10.1007/s42770-026-02090-9

**Published:** 2026-09-10

**Authors:** Kaniz Fatema, Md Mezbaul Bahar, Mallavarapu Megharaj

**Affiliations:** 1 Global Centre for Environmental Remediation (GCER), School of Science, College of Engineering, Science and Environment, The University of Newcastle, ATC Building, Callaghan, NSW 2308 Australia; 2 crc for Contamination Assessment and Remediation of Environment (crc CARE), The University of Newcastle, ATC Building, Callaghan, NSW 2308 Australia

**Keywords:** Cellulolytic, Optimization, Carboxymethyl cellulase (CMCase), Marsupial gut bacteria

## Abstract

Cellulose, the most abundant renewable biopolymer on Earth, represents a key resource for biofuel production but requires efficient enzymatic hydrolysis to overcome its structural resistance. In this study, cellulose-degrading bacteria were isolated from fecal samples of two Australian marsupial herbivores: the koala (*Phascolarctos cinereus*) and the common wombat (*Vombatus ursinus*). Ten bacterial isolates were screened for cellulolytic activity; four strains exhibited substantial hydrolysis zones on carboxymethyl cellulose (CMC) agar and demonstrated elevated activity in fermentation broth. 16 S rRNA gene sequencing identified three isolates as *Streptomyces* spp. (K1, KFK6, KFWH) and one as *Enterobacter* sp. (KFWI). Optimization of cellulase production was conducted by varying incubation periods (24–168 h), pH (3.5–8.5), temperatures (27–50 °C), additives (NaCl, CaCl₂, ZnSO₄, MgSO₄, SDS, EDTA, Tween 80, Triton X-100), carbon sources (glucose, fructose, lactose, sucrose, CMC), and nitrogen sources (yeast extract, ammonium chloride, peptone, urea). K1, KFK6 and KFWI showed maximum CMCase production at 37 °C, whereas KFWH showed maximum production at 40 °C. The optimal pH was 7.5 for K1 and KFWI, and 6.5 for KFK6 and KFWH. Maximum enzyme production occurred at 72 h for K1 and KFWI, 120 h for KFK6, and 96 h for KFWH. These marsupial-derived isolates represent promising candidates for future studies on lignocellulosic biomass hydrolysis and other biotechnological applications.

**Supplementary Information:**

The online version contains supplementary material available at https://doi.org/10.1007/s42770-026-02090-9.

## Introduction

Cellulose is the most abundant renewable biopolymer on Earth and presents significant potential for sustainable biofuel production. However, its degradation is hindered by its high molecular weight, crystalline structure, and insolubility in water [21]. Cellulolysis refers to the hydrolysis of β-1,4-glycosidic bonds, resulting in cellodextrins and glucose monomers, a process typically mediated by synergistic multi-enzyme complexes [16]. A wide range of microorganisms, including bacteria, fungi, and actinomycetes, produce cellulolytic enzymes that facilitate the conversion of lignocellulosic biomass into value-added products such as fermentable sugars, organic acids, biopolymers, and biofuels [8].

Bacterial and actinomycete sources, particularly *Streptomyces* species, are recognized for producing extracellular cellulolytic enzymes involved in lignocellulose degradation [8]. The gut microbiomes of herbivores, including wood-feeding termites, insects, and ruminants, contain specialized cellulolytic communities [10, 22]. In contrast, non-ruminant hindgut fermenters such as Australian marsupials remain relatively underexplored as sources of cellulolytic bacterial isolates, despite evidence that their gut microbiomes are adapted to fibre-rich diets [1].

The koala (*Phascolarctos cinereus*), a specialist folivore, consumes eucalyptus leaves that are rich in lignocellulose and plant secondary compounds. Its digestive system and associated gut microbiota are adapted to this highly fibrous diet, with recent studies identifying microbial functions associated with the degradation of cellulose and other plant structural carbohydrates [1]. Similarly, the common wombat (*Vombatus ursinus*) is a hindgut fermenter that feeds on predominantly on grasses, roots, and other fibrous plant materials, with microbial fermentation in the gastrointestinal tract contributing substantially to the utilization of plant material [3]. Despite these adaptations, koalas and wombats remain relatively underexplored as sources of culturable cellulolytic bacteria. Their fibre-rich diets and dependence on microbial fermentation therefore provide a strong rationale for investigating their gut-associated bacteria for cellulolytic activity.

We hypothesized that fecal samples from koalas and wombats harbour cellulose-degrading bacteria with potential biotechnological applications. Therefore, this study aimed to (i) isolate and characterize cellulolytic bacteria from the feces of these marsupials and (ii) optimize fermentation parameters for cellulase production, with a focus on carboxymethyl cellulase (CMCase) activity. The study provides a culture-based assessment of cellulolytic bacteria from two Australian marsupial herbivores and evaluates the conditions influencing CMCase production by the selected isolates.

## Materials and methods

### Sample collection

Fresh fecal samples from koalas and common wombats were collected aseptically at Blackbutt Reserve Wildlife Sanctuary, Kotara, NSW, Australia, using a sampling approach consistent with previous isolation studies of cellulolytic bacteria from herbivore feces [18]. Samples were placed in sterile 70-mL specimen jars and stored at 4 °C, and processed within 24 h of collection.

### Enrichment and isolation of cellulolytic bacteria

Cellulose-degrading bacteria were enriched using a modified Mandels and Reese medium [13] with the following composition (g/L): peptone, 1.0; urea, 0.3; KH₂PO₄, 2.0; (NH₄)₂SO₄, 1.0; CaCl₂, 0.3; MgSO₄·7 H₂O, 0.3; FeSO₄·7 H₂O, 0.005; MnSO₄·H₂O, 0.016; ZnSO₄·7 H₂O, 0.014; CoCl₂, 0.002; pH adjusted to 7.0. One gram of fecal material from each marsupial was inoculated into 100 mL of this medium in a 250-mL Erlenmeyer flask and incubated at 37 °C with shaking at 150 rpm for 24 h. Subsequently, one milliliter of culture was transferred to fresh medium daily for seven days to enrich cellulolytic populations.

Final enriched cultures were serially diluted in sterile saline (0.85% NaCl), and 100 µL aliquots were spread onto carboxymethyl cellulose (CMC) agar plates, which consisted of enrichment medium supplemented with 1% CMC and 1.5% agar. Plates were incubated at 37 °C for 24 to 48 h. Distinct colonies were purified by repeated streaking on fresh CMC agar until axenic cultures were achieved.

### Qualitative screening for cellulolytic activity

Purified isolates were point-inoculated onto CMC agar plates and incubated at 37 °C for 24 h. Plates were then flooded with 1% (w/v) Congo red for 15 min, followed by destaining with 1 M NaCl [19]. The presence of clear hydrolysis zones around colonies indicated CMCase activity, and zone diameters were measured to assess relative cellulolytic activity. The cellulolytic index (CI) was calculated according to Ferbiyanto et al., [4] as follows:

$$\mathrm{CI}=\;(\mathrm{diameter}\;\mathrm{of}\;\mathrm{hydrolysis}\;\mathrm{zone}-\:\mathrm{diameter}\;\mathrm{of}\;\mathrm{colony})/\mathrm{diameter}\;\mathrm{of}\;\mathrm{colony}$$

where the hydrolysis-zone diameter represents the total diameter of the clear zone, including the bacterial colony.

### Quantitative screening for cellulase production

Selected isolates were cultivated in submerged fermentation using a medium composed of (g/L): CMC, 10 (as substrate); K₂HPO₄, 2.0; MgSO₄, 0.3; peptone, 10; (NH₄)₂SO₄, 2.5; with pH adjusted to 7.0. Twenty milliliters of medium in 100-mL flasks were inoculated with a loopful of bacterial cells from a 24-hour LB agar slant and incubated at 37 °C with shaking at 150 rpm for 24 h. Cultures were centrifuged at 14,000 × g for 10 min at 4 °C, and the cell-free supernatant was used as the crude enzyme source.

CMCase activity was determined using a modified dinitrosalicylic acid (DNS) assay based on the IUPAC cellulase assay described by Ghose [5] and the DNS method of Miller [14]. Briefly, 500 µL of crude enzyme was combined with 500 µL of 1% (w/v) CMC in 0.05 M phosphate buffer (pH 8.0) and incubated at 50 °C for 30 min. These conditions were used consistently for the CMCase activity assay and were distinct from the culture conditions evaluated for optimization of enzyme production. The reaction was terminated by adding 1.5 mL of DNS reagent, followed by boiling for 10 min. Reducing sugars were quantified spectrophotometrically at 540 nm (Shimadzu UV-3600 Plus) using a glucose standard curve. One unit (U) of CMCase activity was defined as the amount of enzyme required to release 1 µmol of reducing sugar (expressed as glucose equivalents) per minute under the assay conditions. All assays were performed in triplicate.

### Molecular identification of isolates

Isolates were cultured in Luria-Bertani (LB) broth (g/L: tryptone, 10; yeast extract, 5; NaCl, 10) at 37 °C for 48 to 72 h with shaking. Cells were collected by centrifugation at 10,000 × g for 1 min, and genomic DNA was extracted using a commercial kit according to the manufacturer’s instructions. The 16 S rRNA gene was amplified by PCR using universal primers 27 F (5’-AGAGTTTGATCMTGGCTCAG-3’) and 1492R (5’-TACGGYTACCTTGTTACGACTT-3’) [6, 17]. PCR reactions were performed in an Applied Biosystems thermocycler using the following program: initial denaturation at 94 °C for 4 min; 30 cycles of 94 °C for 40 s, 55 °C for 1 min, and 72 °C for 1 min 10 s; followed by a final extension at 72 °C for 10 min. Amplicons were purified using the Isolate II PCR and Gel Kit (Bioline) and sequenced at the Ramaciotti Centre for Genomics, Sydney, Australia.

The 16 S rRNA gene sequences were compared with sequences available in GenBank using the BLASTn program of the National Center for Biotechnology Information (NCBI). Sequence similarity and query coverage were recorded from the BLASTn results. Nucleotide sequences were aligned with reference sequences from GenBank, and phylogenetic relationships were assessed using the neighbor-joining method in MEGA X [11]. The robustness of tree topology was evaluated using 1,000 bootstrap replicates. The partial 16 S rRNA gene sequences of the four selected isolates were deposited in GenBank under accession numbers MN685222 (K1), MT397014 (KFK6), MT407644 (KFWH), and MT433926 (KFWI).

### Reducing sugar and saccharification analysis

Reducing sugars generated from enzymatic hydrolysis were quantified using the DNS method [14]. Saccharification percentage was calculated as follows: Saccharification (%) = (Reducing sugars released × 0.9 / Initial substrate concentration) × 100 (Mandels and Sternberg, 1976), where the factor 0.9 accounts for water incorporation during hydrolysis. All analyses were performed in triplicate.

### Protein estimation

Total protein concentration in the cell-free crude enzyme supernatant was determined using the Bradford assay [2], with bovine serum albumin (BSA) as the standard. Five microliters of sample were mixed with 250 µL Bradford reagent (Bio-Rad), incubated for 10 min at room temperature, and absorbance was measured at 595 nm.

### Optimization of cellulase production

#### Effect of fermentation media

Four media were evaluated: Mandels and Reese [13], CMC broth [7], minimal salts medium, and Bushnell-Haas medium [12]. Isolates were pregrown in enrichment medium to exponential phase, then inoculated (5% v/v) into 100 mL of each test medium in 250-mL flasks and incubated at 37 °C with shaking at 150 rpm for 72 h. Supernatants were subsequently assayed for CMCase activity.

#### Effect of incubation period

Cultures in optimized CMC broth were sampled every 24 h from 24 to 168 h, and CMCase activity was measured as previously described.

#### Effects of pH and temperature 

The effects of pH and temperature on CMCase production were evaluated sequentially. For pH optimization, media pH was adjusted using 100 mM acetate buffer (pH 3.5 to 6.5) or phosphate buffer (pH 7.5 to 8.5). Inocula from LB cultures (OD₆₀₀ ≈ 1.0) were added to 50 mL fermentation broth in 250-mL flasks and incubated at 37 °C with shaking at 150 rpm. Following determination of the optimum pH for each strain, the effect of temperature was evaluated at the respective optimum pH over a temperature range of 27–50 °C. CMCase activity was measured after incubation as described in Sect.  "Quantitative screening for cellulase production".

#### Effects of carbon sources, nitrogen sources, and substrate concentration

Carbon sources (glucose, fructose, sucrose, CMC, lactose; 1% w/v) and nitrogen sources (urea, (NH₄)₂SO₄, yeast extract, peptone; 1% w/v) were tested individually in basal fermentation medium. CMC concentrations ranged from 0.5 to 2.5% (w/v). Cultures were incubated under optimal conditions, and CMCase activity was measured.

#### Effects of additives

Metal salts (NaCl, CaCl₂, ZnSO₄, MgSO₄) and surfactants (SDS, Triton X-100, Tween 80), and the chelating agent EDTA were added at concentrations of 5 mM or 10 mM to the optimized fermentation medium. Cultures were incubated under strain-specific optimal conditions, and CMCase activity was assessed. All optimization experiments were conducted in triplicate.

### Statistical analysis

All experiments were performed in triplicate (*n* = 3), and data are presented as mean ± standard deviation (SD). Descriptive statistics were used to summarize the experimental data and variability among replicates. No inferential statistical tests were performed; therefore, differences among treatments or isolates are described as numerical differences and are not interpreted as statistically significant.

## Results and discussion

### Screening and identification of cellulase producing bacteria

**Fig. 1:**
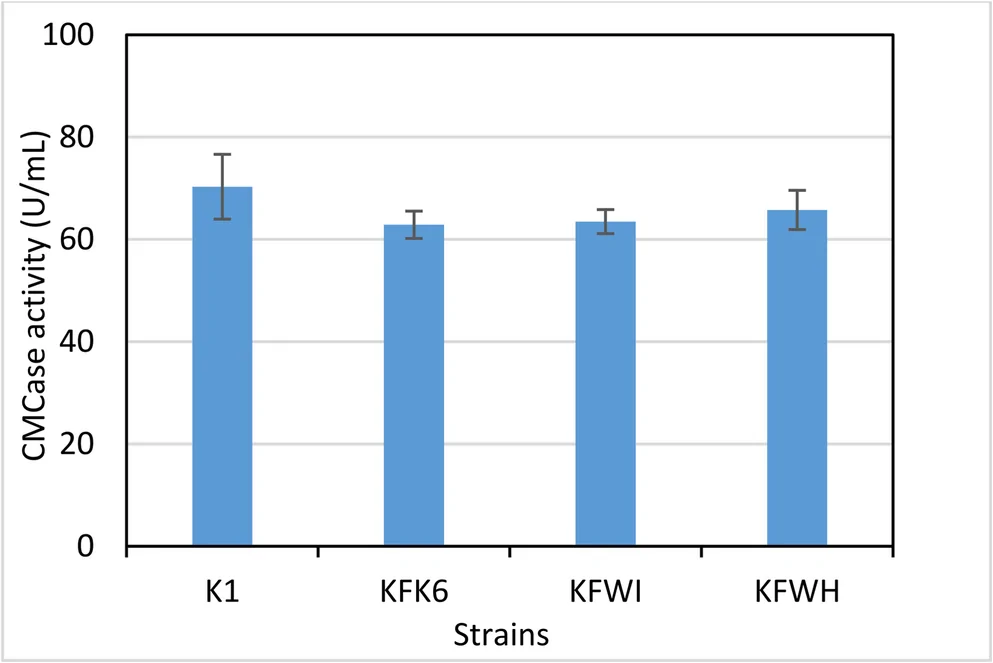
Secondary screening of the selected isolates (K1, KFK6, KFWI and KFWH) for CMCase activity

Ten bacterial isolates obtained from koala and wombat fecal samples demonstrated cellulolytic activity on carboxymethyl cellulose (CMC) agar, as indicated by clear hydrolysis zones following Congo red staining. The diameters of these zones varied, reflecting differences in degradation efficiency (Table 1). Quantitative screening in liquid CMC fermentation medium identified four strains with superior cellulase activity: K1 (70.30 ± 6.33 U/mL), KFK6 (62.86 ± 2.67 U/mL), KFWI (63.49 ± 2.35 U/mL), and KFWH (65.76 ± 3.83 U/mL) (Fig. 1). These differences between plate-based screening and quantitative CMCase activity support the use of liquid enzyme assays for selecting cellulolytic isolates, as hydrolysis-zone size does not necessarily provide a direct measure of enzyme activity.

**Table 1:** Zone of hydrolysis of four isolates

Isolate	Colony diameter (mm)	Hydrolysis zone diameter (mm)	Cellulolytic index (CI)
K1	6.0	17.0	1.83
KFK6	5.5	15.5	1.82
KFWH	6.0	12.0	1.00
KFWI	7.0	13.0	0.86

The partial 16 S rRNA gene sequences obtained for K1, KFK6, KFWH, and KFWI were 1,188, 1,163, 1,314, and 1,368 bp in length, respectively. BLASTn analysis showed 98–100% query coverage and 98.54–100% sequence identity with closely related sequences in GenBank. Phylogenetic analysis supported the identification of K1, KFK6, and KFWH as Streptomyces spp. (GenBank accession numbers MN685222, MT397014, and MT407644, respectively; Fig. S1), while KFWI was identified as Enterobacter sp. (GenBank accession number MT433926; Fig. S2). The predominance of Streptomyces among the selected isolates is consistent with the established cellulolytic capacity of this genus. Recent work has demonstrated that Streptomyces strains can produce multiple cellulolytic enzymes, including endoglucanase, exoglucanase, and β-glucosidase, supporting their role in lignocellulose degradation [8]. The recovery of cellulolytic Streptomyces and Enterobacter isolates from koala and wombat fecal samples therefore supports these marsupial herbivores as potential sources of culturable bacteria with cellulose-degrading activity.

The recovery of cellulolytic *Streptomyces* and *Enterobacter* isolates from these marsupial sources expands the range of culturable bacteria examined for CMCase production from herbivore-associated environments. Importantly, the isolates displayed distinct CMCase production profiles across the culture conditions subsequently evaluated in this study. However, the present data do not establish that these isolates are intrinsically superior to cellulolytic strains from other ecological sources; rather, their interest lies in their underexplored host origin together with their measurable cellulolytic activity and strain-specific responses to production conditions.

### Saccharification of carboxymethyl cellulose by the isolates

**Fig. 2:**
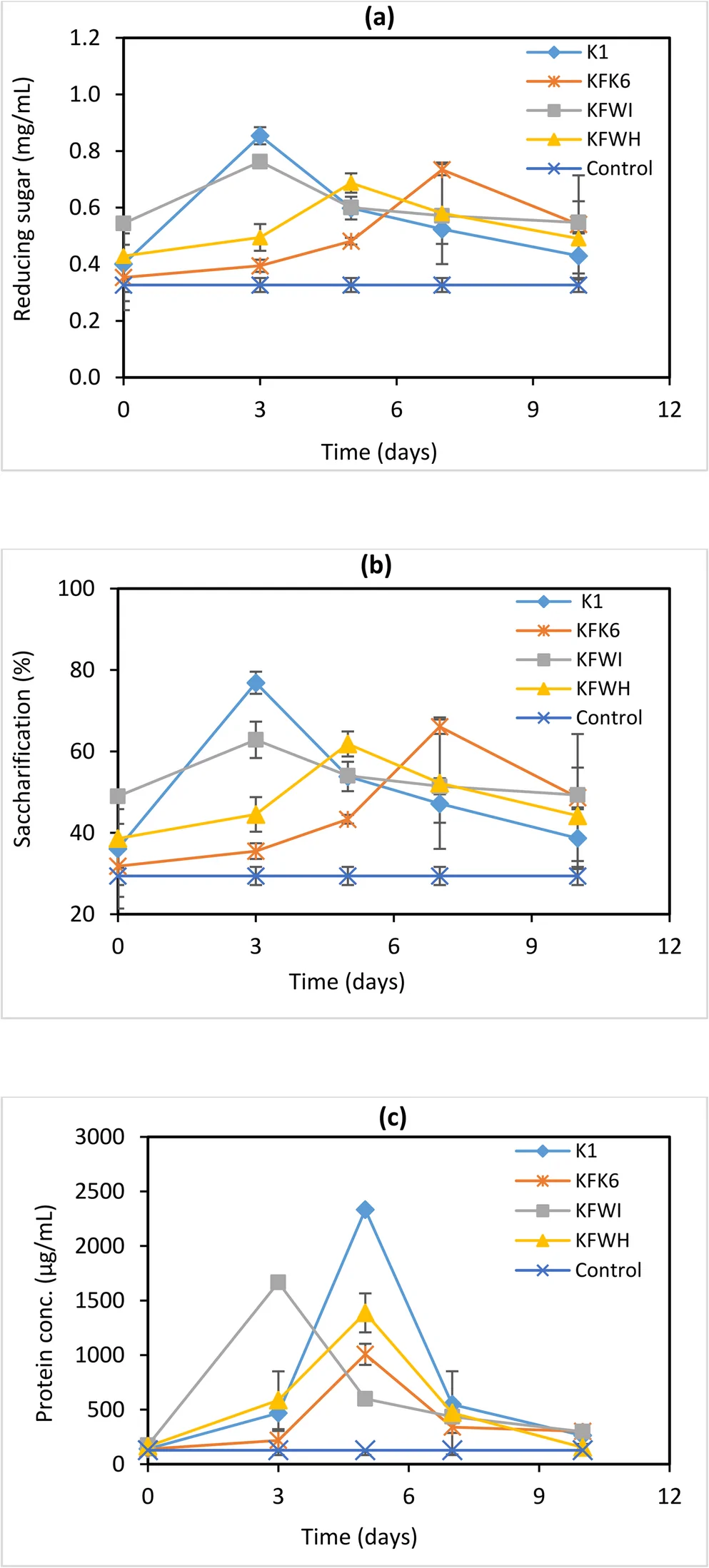
Reducing sugar production (**a**), saccharification (%) (**b**), and total soluble protein concentration in the cell-free culture supernatants (**c**). Data points represent mean (n=3) ± standard deviation

All four strains hydrolyzed carboxymethyl cellulose (CMC), with reducing sugar production varying over the incubation period (Fig. 2a). Strains K1 and KFWI reached maximum reducing sugar concentrations of 0.854 ± 0.03 mg/mL and 0.76 ± 0.02 mg/mL, respectively, on day 3, corresponding to saccharification values of 76.87 ± 2.72% and 62.84 ± 4.47% (Fig. 2b). In contrast, KFK6 reached its maximum reducing sugar concentration (0.73 ± 0.02 mg/mL) and saccharification (66.06 ± 1.77%) on day 7, whereas KFWH reached its maximum values (0.69 ± 0.03 mg/mL and 61.83 ± 3.07%, respectively) on day 5. These results demonstrate strain-specific differences in the timing and extent of CMC hydrolysis.

Total soluble protein concentrations in the cell-free culture supernatants also varied among the isolates and over the incubation period (Fig. 2c). Maximum protein concentrations of 2,883 ± 778 µg/mL (K1), 1,006 ± 96 µg/mL (KFK6), and 1,387 ± 178 µg/mL (KFWH) were observed on day 5, whereas KFWI reached its maximum protein concentration of 2,184 ± 733 µg/mL on day 3. These measurements represent total soluble protein in the cell-free supernatants and were not used as indicators of microbial growth or biomass.

### Effects of media, pH, temperature, and incubation period on CMCase activity

**Fig. 3:**
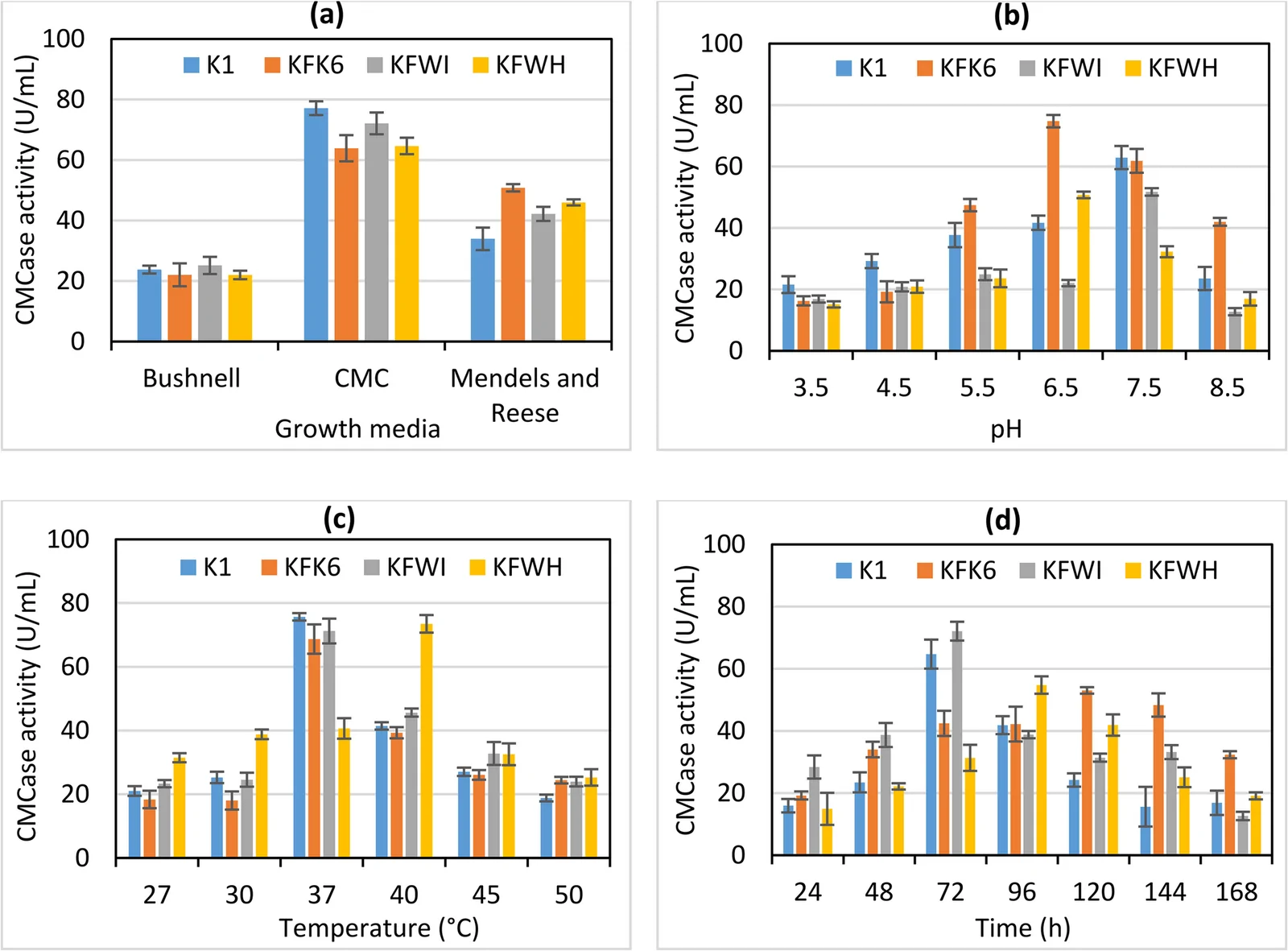
Effects of (**a**) culture medium, (**b**) pH, (**c**) temperature, and (**d**) incubation period on CMCase activity. Data points represent mean (n=3) ± standard deviation

CMC broth yielded the highest CMCase activity for K1 (77.13 ± 2.28 U/mL), KFK6 (63.89 ± 4.32 U/mL), and KFWI (72.10 ± 3.61 U/mL), whereas Mandels and Reese medium was optimal for KFWH (64.64 ± 2.73 U/mL) (Fig. 3a). The variation among media indicates that medium composition influenced CMCase production in a strain-dependent manner. The higher activity of three isolates in CMC broth is consistent with the effectiveness of CMC as a substrate for CMCase production by *Streptomyces*, as demonstrated in recent optimization studies [15, 20].

The optimal pH values varied among isolates: K1 and KFWI exhibited maximum CMCase activity at pH 7.5 (62.93 ± 3.76 U/mL and 51.74 ± 1.22 U/mL, respectively), while KFK6 and KFWH peaked at pH 6.5 (74.76 ± 2.02 U/mL and 50.76 ± 1.08 U/mL, respectively) (Fig. 3b). These results indicate that CMCase production by the four isolates was favored under near-neutral conditions, although the optimum pH was strain dependent. Similar variation in pH requirements has been reported for cellulase-producing *Streptomyces*; for example, optimized cellulase production by *Streptomyces thermodiastaticus* TS4 was reported at an initial pH of 6.0 [20].

The temperature optima were 37 °C for K1, KFK6, and KFWI (75.68 ± 1.17 U/mL, 68.71 ± 4.60 U/mL, and 71.23 ± 3.88 U/mL, respectively) and 40 °C for KFWH (73.49 ± 2.76 U/mL) (Fig. 3c). Thus, all four isolates showed maximum CMCase production within a relatively narrow mesophilic temperature range. The observed variation among isolates is consistent with the strain-dependent influence of temperature on microbial cellulase production [15, 20]. Maximum CMCase activity was observed at 72 h for K1 and KFWI (64.72 ± 4.66 U/mL and 72.10 ± 3.03 U/mL, respectively), at 96 h for KFWH (54.77 ± 2.80 U/mL), and at 120 h for KFK6 (53.03 ± 1.04 U/mL) (Fig. 3d). The different activity profiles demonstrate that the incubation period required for maximum CMCase production was strain dependent. CMCase activity declined after the respective maxima; however, the mechanism responsible for this decline was not investigated in the present study. Variation in optimal fermentation conditions among cellulase-producing *Streptomyces* strains has similarly been reported [20]. 

### Effect of carbon, nitrogen sources and substrate concentrations

**Fig. 4:**
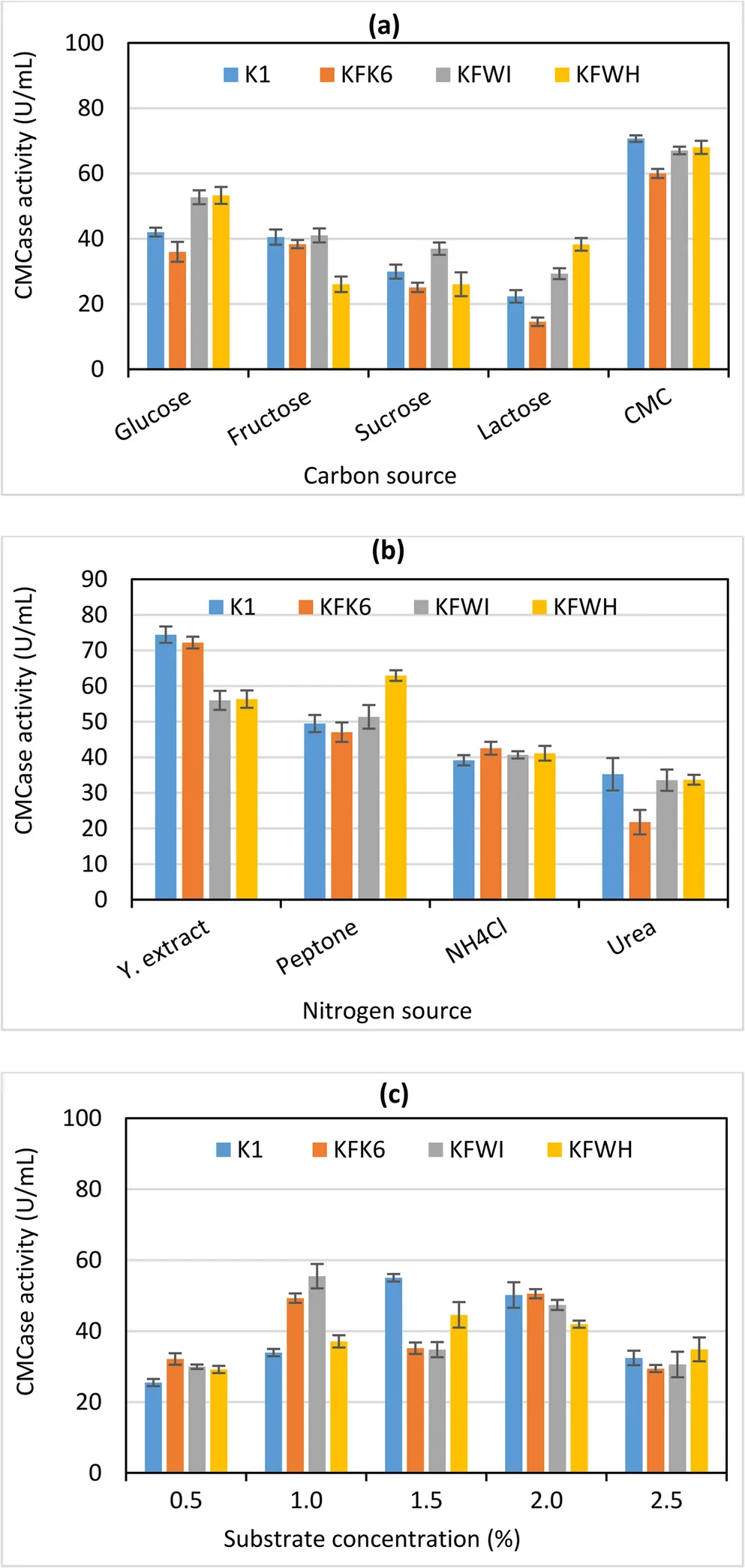
Effects of (**a**) carbon source, (**b**) nitrogen sources, and (**c**) substrate concentration on CMCase activity. Data points represent mean (n=3) ± standard deviation

Carboxymethyl cellulose (CMC) as a carbon source resulted in the highest enzyme activities: 70.66 ± 1.01 U/mL (K1), 59.99 ± 1.40 U/mL (KFK6), 67.04 ± 1.16 U/mL (KFWH), and 68 ± 2.02 U/mL (KFWI) (Fig. 4a). These results indicate that the carbon source influenced CMCase production, with CMC being the most favorable among the substrates tested for all four isolates. The effectiveness of CMC for cellulase production is consistent with recent studies of *Streptomyces*, in which CMC supported or stimulated enzyme production [20, 15].

Among the nitrogen sources tested, yeast extract resulted in the highest CMCase activity for K1, KFK6, and KFWH (74.42 ± 2.30 U/mL, 72.20 ± 1.66 U/mL, and 55.98 ± 2.67 U/mL, respectively), whereas peptone was optimal for KFWI (62.93 ± 1.49 U/mL) (Fig. 4b). These findings demonstrate a strain-dependent response to nitrogen source, with organic nitrogen sources supporting the highest CMCase production under the conditions tested. The influence of nitrogen source on cellulase production has also been demonstrated in recent *Streptomyces* studies, including enhanced production with peptone or tryptone under optimized conditions [15, 20].

CMC concentration also influenced CMCase production (Fig. 4c). K1 and KFWH exhibited maximum activities of 55.02 ± 1.06 U/mL and 44.57 ± 3.60 U/mL, respectively, at 1.5% CMC, whereas KFK6 and KFWI showed maximum activities of 50.52 ± 1.29 U/mL and 55.46 ± 3.44 U/mL, respectively, within the tested range of 1–2% CMC. These results indicate that the CMC concentration supporting maximum CMCase production varied among the isolates. A similar influence of CMC concentration on cellulase production has been reported for *Streptomyces thermodiastaticus* TS4, for which CMC concentration was identified as an important fermentation variable [20].

### Assorted concentrations of different additives on cellulolytic activity

**Fig. 5:**
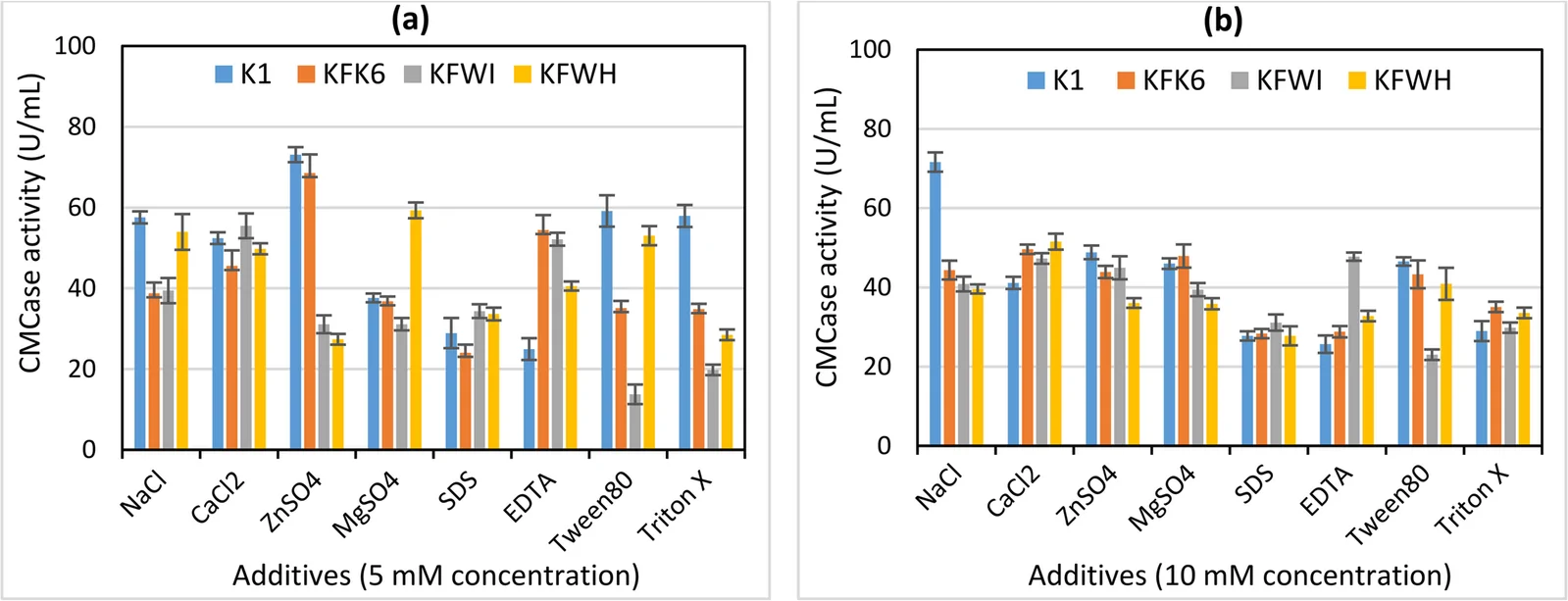
Effects of different additives at concentration of (**a**) 5 mM and (**b**) 10 mM on CMCase activity. Data points represent mean (n=3) ± standard deviation

The effects of metal salts and other additives on CMCase activity varied among the isolates and between the two concentrations tested (Fig. 5a, b). Overall, higher activities were observed at 5 mM than at 10 mM for most additives. At 5 mM, K1 and KFK6 showed their highest CMCase activities in the presence of ZnSO₄ (73.10 ± 1.86 U/mL and 68.54 ± 4.62 U/mL, respectively), whereas KFWI and KFWH showed their highest activities in the presence of CaCl₂ (55.48 ± 3.07 U/mL) and MgSO₄ (59.31 ± 1.95 U/mL), respectively. The effects of metal ions on cellulase activity can vary considerably among enzymes, and differential responses to metal ions and other additives have also been reported for a recently characterized metagenome-derived cellulase [9].

Among the other additives tested, Tween 80 was associated with the highest CMCase activities for K1 and KFWH (59.16 ± 3.89 U/mL and 53.03 ± 2.37 U/mL, respectively), whereas KFK6 and KFWI showed their highest activities in the presence of EDTA (54.44 ± 3.69 U/mL and 52.16 ± 1.60 U/mL, respectively). The effects of surfactants and chelating agents on cellulase activity are also enzyme dependent. Recent studies have demonstrated responses of cellulases to non-ionic surfactants and other additives [9], while Tween 80 has been reported to promote CMCase production by *Streptomyces* sp. CSMPJR101 [15]. The strain-specific responses observed here indicate that the effects of additives on CMCase activity depend on both the isolate and additive concentration.

Although the maximum CMCase activities observed during optimization were generally comparable to those obtained during the initial quantitative screening, the optimization experiments identified distinct strain-specific conditions that favored CMCase production. The isolates differed in their preferred medium, pH, temperature, incubation period, nitrogen source, CMC concentration, and response to additives. Thus, the primary contribution of the optimization experiments was the identification of favorable culture conditions for each isolate rather than a substantial increase in CMCase activity over the initial screening values. Further multivariable optimization would be required to determine whether combining these conditions can significantly increase enzyme production.

## Conclusion

Four cellulolytic bacterial strains were isolated and characterized from the feces of two Australian marsupials, the koala and common wombat. The isolates K1, KFK6, and KFWH were identified as *Streptomyces* spp., while KFWI was identified as *Enterobacter* sp. All four isolates exhibited CMCase activity, with the highest activity observed for K1 (77.13 ± 2.28 U/mL). The effects of culture medium, pH, temperature, incubation period, carbon and nitrogen sources, CMC concentration, and selected additives varied among the isolates, demonstrating strain-specific conditions associated with CMCase production. These findings indicate that marsupial-derived bacteria represent a potential source of cellulolytic enzymes. However, the present study was limited to CMCase activity using CMC as a model substrate. Further studies should characterize additional cellulolytic enzymes, evaluate enzyme performance using raw or pretreated lignocellulosic biomass, and assess hydrolysis efficiency before the suitability of these isolates for biofuel or other industrial applications can be established. 

## Supplementary Information

Below is the link to the electronic supplementary material.

Supplementary Material 1
