# Supplementary material for: Cellulolytic potential of bacteria isolated from Australian marsupial herbivores
Source: Braz J Microbiol. 2026 Sep 10;57(1):265. doi: 10.1007/s42770-026-02090-9 (PMC13562478; doi:10.1007/s42770-026-02090-9)
Supplement: Supplementary file 1 [file 42770_2026_2090_MOESM1_ESM.docx]

*Supplementary Information*

**Cellulolytic Potential of Bacteria Isolated from Australian Marsupial Herbivores**

**Fig. S1.** Neighbor-Joining tree based on the comparison of 16s rRNA gene sequences showing the relationships among *Streptomyces* sp. strains. The accession numbers of the strains are given in parenthesis.

**Fig. S2.** Neighbor-Joining tree based on the comparison of 16s rRNA gene sequences showing the relationships among *Enterobacter* sp. strains. The accession numbers of the strains are given in parenthesis.
